# Supplementary material for: The relationships between toad behaviour, antipredator defences, and spatial and sexual variation in predation pressure
Source: PeerJ. 2022 Feb 17;10:e12985. doi: 10.7717/peerj.12985 (PMC8858576; doi:10.7717/peerj.12985)
Supplement: Supplemental Information 1 [file peerj-10-12985-s001.docx]

**Behavioral Ecology and Sociobiology**

**Supplementary Material**

**The relationships between toad behaviour, antipredator defences, and spatial and sexual variation in predation pressure**

Francisco Javier Zamora-Camacho

Departamento de Sistemas Físicos, Químicos y Naturales. Universidad Pablo de Olavide, Carretera de Utrera km 1, 41080, Seville, Spain.

Departamento de Biodiversidad, Ecología y Evolución, Facultad de Ciencias Biológicas, Universidad Complutense de Madrid, C/José Antonio Novais 12, 28040 Madrid, Spain.

e-mail: zamcam@ugr.es

Table S1 – Correlation matrix between behavioural traits measured. Indicated are *r-* and *P*-values.

|  | **Number of squares visited** | **Number of square visits** | **Internal squares visited ratio** | **Internal square visits ratio** | **Activity time** | **Time until the first move** |
| --- | --- | --- | --- | --- | --- | --- |
| **Number of squares visited** |  | *r* = 0.874  *P* < 0.001 | *r* = -0.528  *P* < 0.001 | *r* = -0.487  *P* < 0.001 | *r* = 0.685  *P* < 0.001 | *r* = -0.189  *P* = 0.078 |
| **Number of square visits** | *r* = 0.874  *P* < 0.001 |  | *r* = -0.425  *P* < 0.001 | *r* = -0.406  *P* < 0.001 | *r* = 0.714  *P* < 0.001 | *r* = -0.149  *P* = 0.166 |
| **Internal squares visited ratio** | *r* = -0.528  *P* < 0.001 | *r* = -0.425  *P* < 0.001 |  | *r* = 0.919  *P* < 0.001 | *r* = -0.391  *P* < 0.001 | *r* = -0.072  *P* = 0.507 |
| **Internal square visits ratio** | *r* = -0.487  *P* < 0.001 | *r* = -0.406  *P* < 0.001 | *r* = 0.919  *P* < 0.001 |  | *r* = -0.357  *P* < 0.001 | *r* = -0.080  *P* = 0.461 |
| **Activity time** | *r* = 0.685  *P* < 0.001 | *r* = 0.714  *P* < 0.001 | *r* = -0.391  *P* < 0.001 | *r* = -0.357  *P* < 0.001 |  | *r* = -0.156  *P* = 0.148 |
| **Time until the first move** | *r* = -0.189  *P* = 0.078 | *r* = -0.149  *P* = 0.166 | *r* = -0.072  *P* = 0.507 | *r* = -0.080  *P* = 0.461 | *r* = -0.156  *P* = 0.148 |  |

Table S2 – Correlation matrix between antipredator defences measured. Indicated are *r-* and *P*-values.

|  | **Body mass** | **Parotoid gland contrast** | **Parotoid gland relative area** | **Relative sprint speed** |
| --- | --- | --- | --- | --- |
| **Body mass** |  | *r* = 0.108  *P* = 0.317 | *r* = 0.068  *P* = 0.528 | *r* = -0.076  *P* = 0.484 |
| **Parotoid gland contrast** | *r* = 0.108  *P* = 0.317 |  | *r* = -0.006  *P* = 0.956 | *r* = -0.004  *P* = 0.967 |
| **Parotoid gland relative area** | *r* = 0.068  *P* = 0.528 | *r* = -0.006  *P* = 0.956 |  | *r* = 0.364  *P* < 0.001 |
| **Relative sprint speed** | *r* = -0.076  *P* = 0.484 | *r* = -0.004  *P* = 0.967 | *r* = 0.364  *P* < 0.001 |  |

Table S3 – Results of the PCA including the behavioural traits measured that were correlated (number of squares visited, number of square visits, internal squares visited ratio, internal square visits ratio, and activity time; Table S3a) and the PCA including the antipredator defences measured that were correlated (parotoid gland relative area and relative sprint speed; Table S3b).

|  | | **PCa** | **PCb** | **PCc** | **PCd** | **PCe** |
| --- | --- | --- | --- | --- | --- | --- |
| **Eigenvalue** | | 3.323098 | 1.126300 | 0.351899 | 0.122510 | 0.076193 |
| **Percentage of variance explained** | | 66.46196 | 22.52600 | 7.03798 | 2.45020 | 1.52386 |
| **Coordinate correlations with variables** | **Number of squares visited** | 0.850348 | -0.412439 | 0.218779 | 0.236779 | -0.053614 |
|  | **Number of square visits** | 0.888227 | -0.291613 | 0.259492 | -0.235165 | 0.058113 |
|  | **Internal squares visited ratio** | -0.766180 | -0.610002 | 0.023076 | -0.087390 | -0.180821 |
|  | **Internal square visits ratio** | -0.790488 | -0.577923 | 0.029572 | 0.055817 | 0.192729 |
|  | **Activity time** | 0.774053 | -0.406274 | -0.485069 | -0.019765 | 0.010054 |

Table S3a.

|  | | **PC1** | **PC2** |
| --- | --- | --- | --- |
| **Eigenvalue** | | 1.363980 | 0.636020 |
| **Percentage of variance explained** | | 68.19902 | 31.80098 |
| **Coordinate correlations with variables** | **Parotoid gland relative area** | 0.825827 | 0.563924 |
|  | **Relative sprint speed** | 0.825827 | -0.563924 |

Table S3b.
